# Automatic detection advantage of network information among Internet addicts: behavioral and ERP evidence

**DOI:** 10.1038/s41598-018-25442-4

**Published:** 2018-06-12

**Authors:** Jinbo He, Yang Zheng, Yufeng Nie, Zongkui Zhou

**Affiliations:** Key Laboratory of Adolescent Cyberpsychology and Behavior of Ministry of Education; Key Laboratory of Human Development and Mental Health of Hubei Province; School of Psychology, Central China Normal University, Wuhan, China

## Abstract

Converging evidence has proved the attentional bias of Internet addicts (IAs) on network information. However, previous studies have neither explained how characteristics of network information are detected by IAs with priority nor proved whether this advantage is in line with the unconscious and automatic process. To answer the two questions, this study aims to investigate whether IAs prioritize automatic detection of network information from the behavior and cognitive neuroscience aspects. 15 severe IAs and 15 matching healthy controls were selected using Internet Addiction Test (IAT). Dot-probe task with mask was used in the behavioral experiment, while deviant-standard reverse oddball paradigm was used in the event-related potential (ERP) experiment to induce mismatch negativity (MMN). In the dot-probe task, when the probe location appeared on the Internet-related picture’s position, the IAs had significantly shorter reaction time than do the controls; in the ERP experiment, when Internet-related picture appeared, MMN was significantly induced in the IAs relative to the controls. Both experiments show that IAs can automatically detect network information.

## Introduction

In the 1980s, computer and network technology has combined with and gradually integrated into human life. Then came the Internet era. The convenience, anonymity, and escapism of the network provide people with convenience, comfort, and happiness but trap many into indulgence or dependence and even Internet addiction^1–3^. Internet has become an indispensable element and platform in people’s daily life and work, and the severity of Internet addiction and the quantity of Internet addicts (IAs) are increasing daily. Therefore, Internet addiction has been included in the current edition of the Diagnostic and Statistical Manual of Psychiatric Disorders (DSM-V)^4^. Similar to other addictions, one of the core symptoms of Internet addiction is salience, that is, IAs consider Internet as the main part of their lives and even avoid or ignore other normal social work, thereby leading to negative consequences in the psychological and social aspects and work difficulties. This salience is first manifested by the attentional bias of IAs to network information at the cognitive level^5–8^.

Attentional bias means that addicts are more inclined to show attentional processing advantage over addiction-related cues^9^. Several studies have investigated the attentional bias to Internet-related cues from the behavior level^6,7,10^. For example, Metcalf *et al*.^6^ used the addiction Stroop paradigm and found that online game addicts (OGAs) show a longer response time to game-related and negative words than does the control group, suggesting that the processing of addictive words interferes with the reaction to colors. The results show that IAs put more attentional resourses to network information. Some studies have also investigated the problem from the neural level^8,11^. For instance, Zhang *et al*.^8^ performed MRI scans on 19 IAs in the Stroop task and discovered that IAs respond more quickly to Internet-related words than do healthy individuals. Network words induce higher activation in the inferior parietal lobule, middle occipital gyrus, and dorsolateral prefrontal cortex at the same time than do non-network words. These brain regions are associated with attentional processing^12^, suggesting that IAs put a large amount of neural resources on network-related words. These findings prove that IAs show attentional bias toward network-related cues on behavior and cognitive neural processing.

Why addicts show attentional bias toward addictive cues? There are two main theoretical explanations. One is cognitive processing theory proposed by Tiffany^13^. The cognitive processing theory states that frequent addictive behaviors may form an automatic action schema, under which addicts process addictive stimulus automatically and seek for addictive behavior unconsciously. Therefore, the attentional bias of addicts is mostly automatic and unconscious and independent of subjective craving and intentional addictive behaviors. The other one is incentive sensitization model proposed by Robinson and Berridge^14^. According to this model, long-term addictive stimulation changes the brain system function related to the reward circuit, which is a dopamine functional projection area that consists of the ventral mesencephalon to ventral striatum in the ventral tegmental area, amygdala, diaphragmatic nucleus, prefrontal cortex and cingulate cortex (Nacc). This area can gradually raise the sensitivity of the Nacc motivational center to addiction-related cues, that is, neural sensitization. Neural sensitization leads to the psychological and implicit characterization of addiction-related cues through addiction salience and causes a pathological desire for addictive behavior^15^. From these two theories, it is not hard to draw a conclusion that, when it comes to the attentional bias, the researchers above emphasized the automatic and unconscious processing of addicts toward addiction-related cues. However, the empirical evidence was based on experimental tasks from conscious processing and thus lacked direct experimental verification of the unconscious and automatic processing. If the automatic detection advantage of IAs on characteristics of network information can be proved under the unconscious state, we will not only enrich the study of the area, but also provide direct evidence of automatic processing advantage for the unconscious bias theories above. In this sense, it is very important and necessary to explore IAs’ automatic detection of network information.

Considering that previous studies of attentional bias were mainly conducted from two levels of behavior and neuroscience, in order to provide corresponding evidence, our research was also carried out from these two levels. Behavioral studies on automatic detection advantage for a certain type of stimulus usually use visual probe task^16–18^. The visual probe task is also called the dot-probe task, in which subjects are asked to determine the probe location after paired stimuli are presented (addiction and neutral stimuli). The probe appears randomly in the position of a previous word. Subjects’ reaction to the probe will be influenced by previous stimulus attributes. If subjects’ reaction time is shorter when the probe appears in the position previously noticed by the subject, there is attentional bias to the stimuli^19^. Mogg and Bradley^16^ suggested that, with this paradigm, brief stimulus presentation (≤30 ms) and masking could explore the individual’s unconscious processing toward related stimuli.

At the level of cognitive neuroscience, Mismatch negativity (MMN) is considered as one of generally accepted electrophysiological index which reflects the automatic detection of changes in stimulus characteristics. MMN was found and named by Näätänen^20^, which is an automatic processing product of nerve cells in the unconscious state. MMN is usually evoked by passive oddball paradigm. The experiment block consists of a large probability standard stimulus and a small probability deviant stimulus, and the ERPs are obtained by subtracting the standard stimulus ERPs with the deviant ones^21^. In the beginning, researches on MMN were limited to auditory modality. In recent years, several studies have discovered that the automatic detection of changes of visual channel stimulus characteristics can also produce MMN. These visual stimulus features include not only simple physical attributes, such as color^22^, position^23,24^, and direction of motion^25^ but also complex class attributes^26^, such as emotional faces^27,28^, opposite hands^29^, and gender faces^30^. Besides, visual MMN could also be used in the study of perceptual processing ability of patients with various neurological disorders, such as schizophrenia^31,32^, depression^33–35^ and long-term amphetamine abusers^36^ (for review see Kremláček, *et al*.^37^).

Visual channel is an important sensory channel for human beings. For IAs, the network information that causes their addiction behaviors mainly comes from the visual channel input. Therefore, this study combined behavior experiments with ERP using dot-probe and the oddball paradigm to integrally investigate whether IAs have automatic detection advantage toward the characteristics of visual network information. We choose realistic network features with high ecological validity as stimulus material. In the behavioral dot-probe paradigm, short stimulus presentation and masking are used to induce unconscious and automatic processing. In the ERP experiment, the deviant-standard reverse oddball paradigm is used to eliminate the effects of stimulus’ physical attributes^38,39^. Moreover, color change is used as a masking stimulus^26^, and unconscious and automatic processing conditions are induced by the judgement of “+” in the central visual field^38,39^. Our hypothesis is that, in behavioral dot-probe experiments, IAs have a short detection response to Internet-related picture stimuli. And in the ERP experiment, the amplitude of MMN of the IAs induced by color change of Internet-related pictures is higher than that of the control group.

## Results

### Behavioral Data

The raw data from E-prime 2.0 was converted and processed using Excel 2016 and SPSS 21.0. Reaction errors and reactions plus or minus three standard deviations were eliminated. Then, mixed ANOVA with 2 (IAs, control group) ×2 pictures (the probe appeared at the same location of the Internet-related pictures, the probe appeared at the opposite location of the Internet-related pictures) was conducted on the effective data of 2 groups of 30 subjects. The results are shown in Table 1.

**Table 1:** Mean reaction time (ms) between groups interfered by image types.

	IAs(M ± SD)	Control group(M ± SD)
at the same location	399.88 ± 14.26	389.98 ± 6.87
at the opposite location	372.76 ± 26.08	389.84 ± 8.43

**Figure 1:**
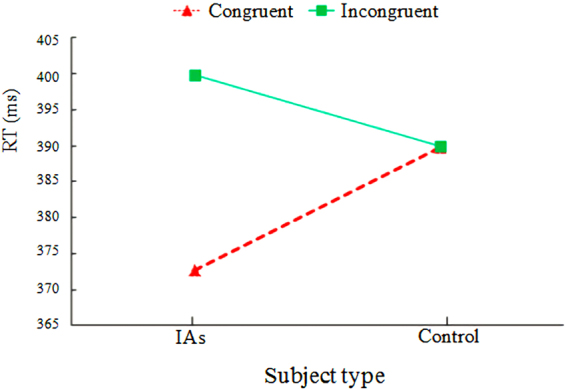
Interaction between the subject type and the location of the probe point. IAs, Internet addicts group; Control, control group; Congruent, the probe dot appeared at the same location of the Internet-related pictures; Incongruent, the probe dot appeared at the opposite location of the Internet-related pictures.

The main effect of the location of the probe dot was significant (*F*_1,28_ = 29.33, *p* < 0.01, *η*^2^ = 0.51). The reaction time when the probe dot showed in the congruent position with the Internet-related pictures [381.30 ms ± 17.25 ms (M ± SD)] was significantly shorter than that in the incongruent position (394.93 ± 10.57). The main effect of the type of the subjects was not significant (*F*_1, 28_ = 0.47, *p* = 0.49). The analysis of variance showed that subject type and probe location had significant interactive effects (Fig. 1) with *F*_1, 28_ = 28.73, *p* < 0.01, and *η*^2^ = 0.50. The comparison of the groups through a simple effect analysis showed that, when the probe appeared at the same location as the Internet-related pictures, a significant difference was observed between the IAs and the control group with *F*_1, 28_ = 5.82, *p* < 0.05, and *η*^2^ = 0.17. The reaction time of IAs was significantly less than that of the control group. When the probe appeared at the opposite location of the Internet-related pictures, a significant difference was observed between the two groups with *F*_1, 28_ = 5.87, *p* < *0.05*, and *η*^2^ = *0.17*. The reaction time of IAs was significantly longer than that of the control group. The comparison within groups showed that the IAs had a significant difference when the probe appeared at a different location with *F*_1, 14_ = 39.98, *p* < 0.01, and *η*^2^ = 0.74. When the location of the probe and the Internet-related pictures were congruent, the reaction time was significantly less than that of incongruent images. However, the reactions between the control groups under the two conditions showed no significant difference.

### ERP Data

We calculated the accuracy of the response of the subjects to the change in “+” size to investigate the concentration of subjects. The results showed that the accuracy of the two groups in four blocks were higher than 90%, indicating that all the subjects followed the experimental guidelines that focus on the changing size of “+” but ignore the image recognition process on both sides.

**Figure 2:**
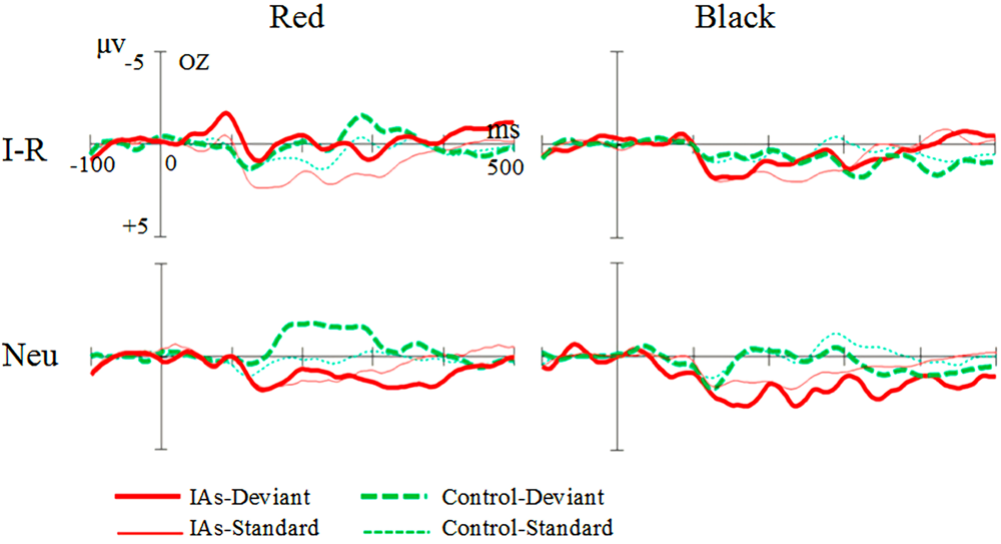
Comparison of groups of two types and two color pictures between the average ERP groups. I-R, Internet-related pictures; Neu, neural pictures; IAs-Deviant, deviant ERPs in IAs; IAs-Standard, standard ERPs in IAs; Control-Deviant, deviant ERPs in control group; Control-Standard, standard ERPs in control group.

Taking Oz as the representative electrode to observe the ERPs’ waveforms of four pictures in the IAs and the control group, Fig. 2 shows that a clear difference existed between the deviant and standard ERPs of each image and that the deviant ERPs were significantly negative. The ERPs of the IAs (red) and the control group (green) significantly differed. The difference between the ERPs of the standard and deviant stimuli was greater in the IA group than in the control group on the red Internet-related picture. However, on the red neutral picture, the difference of ERPs between the control group for the standard and deviation stimuli was significantly greater than that of the IA group.

**Figure 3:**
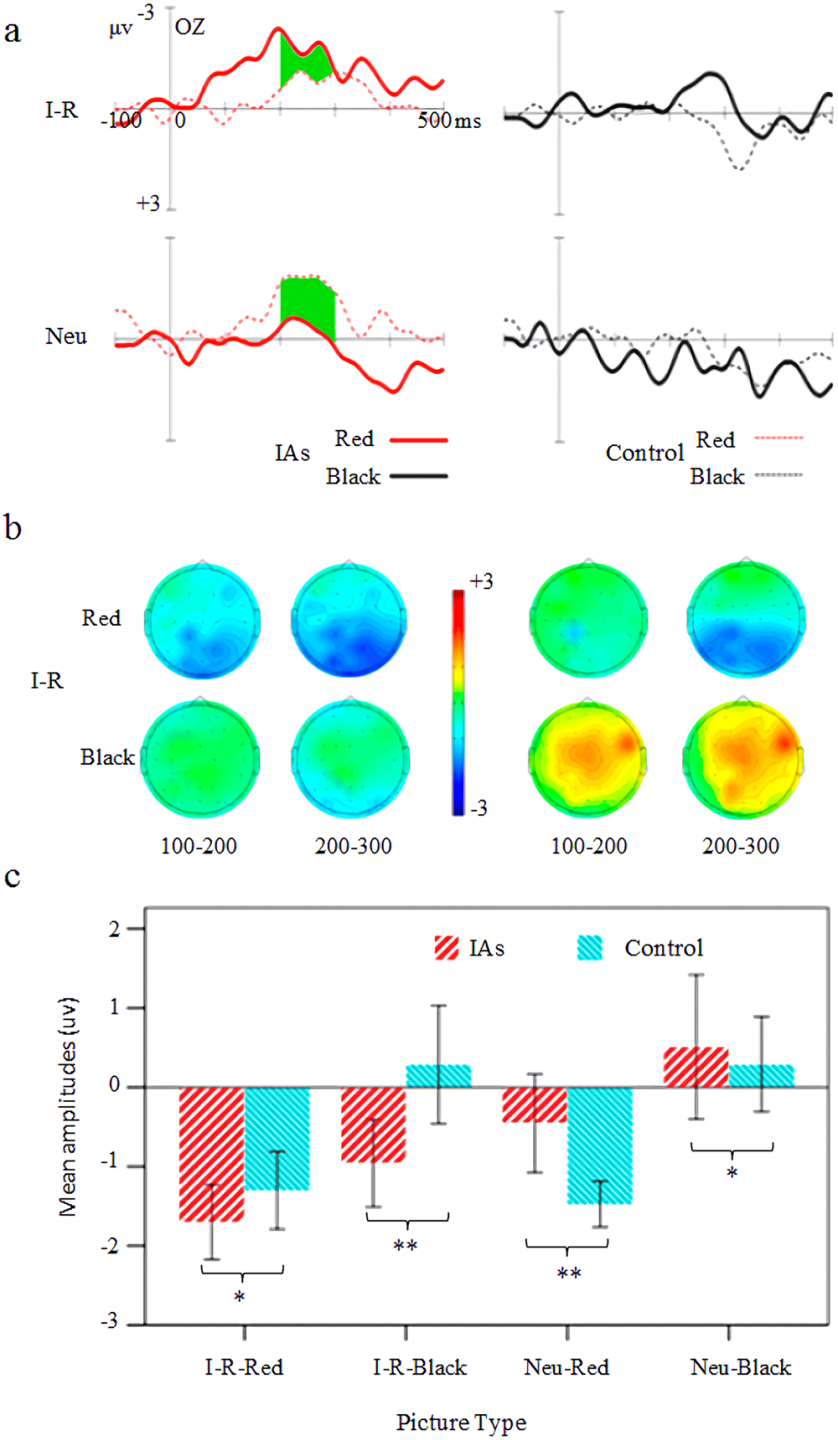
Comparison among groups of MMN between two types and color pictures. (**a**) Comparison of MMN waveforms on OZ electrodes between four types of pictures. I-R, comparison of Internet-related pictures. Neu, comparison of neural pictures. (**b**) Comparison of the MMN BEAMs between two color types. (**c**) Comparison of the mean MMN amplitudes between four types of pictures.

Two groups with 30 subjects of MMN were obtained by subtracting the standard ERPs from each of the four pictures’ respective deviant ERPs, as shown in Fig. 3a,b and c (a, b, and c are waveforms, topographic maps, and statistical bars). Referring to the statistical method introduced in Section 2.2.3, O1 and O2 were used as the representative electrodes of the occipital region, PO5 and PO6 were used as the representative electrode of the temporal occipital region, and the average amplitudes of these MMNs at 200–300 ms were analyzed by MANOVAs.

The Oz analysis is shown in Fig. 3. The picture type exerted a significant effect with *F*_1, 28_ = 12.87, *p* < 0.01, and *η*^2^ = 0.32. The amplitude of the Internet-related pictures (−0.92 ± 0.90) was significantly more negative than that of the neural pictures (−0.28 ± 0.81). The color type had a significant effect with *F*_1, 28_ = 33.22, *p* < 0.01, and *η*^2^ = 0.54. The amplitude of the red pictures (−1.23 ± 0.68) was significantly more negative than that of the black pictures (−0.03 ± 1.03). However, the main effect of the hemisphere (left, right) (*F*_1, 28_ = 0.02, *p* = 0.89) and the subject type (*F*_1, 28_ = 1.07, *p* = 0.25) were insignificant. The interaction between the picture and subject types was significant with *F*_1, 28_ = 16.53, *p* < 0.01, and *η*^2^ = 0.37. A simple effect analysis revealed that, as for the difference between groups, significant difference in the amplitude of the Internet-related pictures between the IAs and the control group was observed with *F*_1, 28_ = 7.63, p < 0.01, and *η*^2^ = 0.21. The amplitude of the IAs (−1.33 ± 0.58) was significantly more negative than that of the control group (−0.51 ± 0.99). A significant difference in the amplitude of the neutral pictures between the IAs and the control group was observed with *F*_1, 28_ = 4.97, p < 0.05, and *η*^2^ = 0.15. The amplitude of the control group (−0.59 ± 0.55) was significantly more negative than that of the IAs (0.03 ± 0.92). As for the difference within groups, significant differences in the amplitude of the IAs between the neutral and Internet-related pictures were observed with *F*_1, 14_ = 25.85, *p* < 0.01, and *η*^2^ = 0.65. The amplitude of the Internet-related pictures (−1.33 ± 0.58) was significantly more negative than that of the neutral pictures (0.03 ± 0.92). No significant difference was observed in the amplitude of the control group between the Internet-related and neutral pictures. But the two-way interaction effects between subject type and color type (*F*_1, 28_ = 2.32, *p* = 0.11), the subject type and hemisphere (left, right) (*F*_1, 28_ = 1.15, *p* = 0.29), picture type and color type(*F*_1, 28_ = 0.38, *p* = 0.54), picture type and hemisphere (left, right) (*F*_1, 28_ = 0.53, *p* = 0.47), and color type and hemisphere (left, right) (*F*_1, 28_ = 1.14, *p* = 0.28) were insignificant. The three-way interaction effects among subject type, picture type and color type (*F*_1, 28_ = 0.03, *p* = 0.87), subject type, picture type and hemisphere (left, right) (*F*_1, 28_ = 0.01, *p* = 0.99), and picture type, color type and hemisphere (left, right) (*F*_1, 28_ = 1.97, *p* = 0.17) were insignificant. The four-way interaction effect among subject type, picture type, color type and hemisphere (left, right) (*F*_1, 28_ = 0.10, *p* = 0.76) was insignificant.

The occipital-temporal electrode site analysis showed that the main effect of the picture type was significant with *F*_1, 28_ = 7.81, *p* < 0.01, and *η*^2^ = 0.22. The amplitude of the Internet-related pictures (−1.12 ± 1.01) was significantly more negative than that of the neutral pictures (−0.46 ± 1.08). The main effects of color type was significant with *F*_1, 28_ = 20.96, *p* < 0.01, and *η*^2^ = 0.43. The amplitude of the red pictures (−1.34 ± 0.80) was significantly more negative than that of the black pictures (0.23 ± 1.17). However, the main effect of the hemisphere (left, right) (*F*_1, 28_ = 0.02, *p* = 0.89) and the subject type (*F*_1, 28_ = 0.17, *p* = 0.69) were insignificant. The interaction between the picture and subject types was significant with *F*_1, 28_ = 11.76, *p* < 0.01, and *η*^2^ = 0.30. A simple effect analysis revealed that, as for the difference between groups, a significant difference was observed in the amplitude of the Internet-related pictures between the IAs and the control group with *F*_1, 28_ = 4.81, p < 0.05, and *η*^2^ = 0.15. The amplitude of the IAs (−1.49 ± 0.78) was significantly more negative than the control group (−0.74 ± 1.09). A significant difference was observed in the amplitude of the neutral pictures between the IAs and the control group with *F*_1, 28_ = 5.51, p < 0.05, and *η*^2^ = 0.16. The amplitude of the control group (−0.89 ± 0.49) was significantly more negative than that of the IAs (−0.03 ± 1.33). As for the difference within groups, significant differences were observed in the amplitude of the IAs between the neutral and Internet-related pictures with *F*_1, 14_ = 12.29, *p* < 0.01, and *η*^*2*^ = 0.47. The amplitude of the Internet-related pictures (−1.49 ± 0.78) was significantly more negative than that of the neutral pictures (−0.02 ± 1.33). No significant difference was observed in the amplitude of the control group between the Internet-related and neutral pictures. The two-way interaction effects between subject type and color type (*F*_1, 28_ = 3.66, *p* = 0.07), the subject type and hemisphere (left, right) (*F*_1, 28_ = 0.09, *p* = 0.76), picture type and color type(*F*_1, 28_ = 0.15, *p* = 0.70), picture type and hemisphere (left, right) (*F*_1, 28_ = 0.01, *p* = 0.99), and color type and hemisphere (left, right) (*F*_1, 28_ = 1.67, *p* = 0.21) were insignificant. The three-way interaction effects among subject type, picture type and color type (*F*_1, 28_ = 0.19, *p* = 0.66), subject type, picture type and hemisphere (left, right) (*F*_1, 28_ = 0.06, *p* = 0.81), and picture type, color type and hemisphere (left, right) (*F*_1, 28_ = 1.32, *p* = 0.26) were insignificant. The four-way interaction effect among subject type, picture type, color type and hemisphere (left, right) (*F*_1, 28_ = 0.04, *p* = 0.83) was insignificant.

## Discussion

Considering the behavioral level, dot-probe with masking task was used in Experiment 1 to investigate whether there is automatic detection advantage of the Internet-related pictures among the IAs. The results showed that the response time of the IAs was shorter than that of the control group when the probe was consistent with the Internet-related picture, which suggested that the IAs might prioritize Internet-related pictures even in a weak state of consciousness. Therefore, when the subsequent probe dot appeared at the same location, a fast position judgment could be made. By contrast, when the probe was inconsistent with the Internet-related picture, a long response was required owing to the lack of early attentional resources to this location. The experimental tasks of this study drew lessons from Mogg *et al*.^16,40^ and their study of individuals with high trait anxiety, in which they used a dot-probe task of a brief presentation of stimuli and masking to regulate the unconscious processing. Their study revealed that individuals with high trait anxiety have an automatic detection advantage toward threat stimuli. Accordingly, the results of this study suggested that IAs had an automatic detection advantage toward Internet-related pictures, that is, IAs would pay attention to Internet-related pictures before they realize the content of the stimulating material.

Considerable research on addiction-related cues has similar results. A modified dot-probe paradigm was used by Yan *et al*.^41^. They controlled the conscious processing by increasing the cognitive load and using masking and found that cigarette addicts have a preattentive bias toward cigarette-related cues. Some studies have explored the stage of attentional bias occurrence (attentional orienting or attention maintenance) to addiction-related cues by changing the presentation time of the dot-probe paradigm. The results indicated that the addicts are inclined to show bias of early visual orientation toward addictive cues^42–45^. Other studies used eye movement techniques to investigate this problem and found similar findings. Rosse *et al*.^46^ found that cocaine addicts in the withdrawal period show an initial orienting advantage to cocaine-related cues during short-term gaze (<50 ms), which disappears during the long-term gaze (>50 ms). This result showed that, although they have the will to control the attentional bias to addiction-related cues after the withdrawal training, they still cannot control the automatic processing advantage to addiction-related cues. Subsequent studies of eye movements, such as alcohol addicts on alcohol-related cues^47,48^, obesities on food-related cues^49,50^, gambling addicts on gambling-related cues^51^, and heroin addicts on heroin-related cue^52^, have reached similar conclusions. These studies have suggested that addicts may have an unconscious automatic attentional processing advantage to addictive cues.

Why do IAs have automatic detection advantage toward Internet-related pictures? First, according to Tiffany’s automatic action schema theory^13^, frequent network-related behavior can make the brain of IAs form an automatic action schema through memory, thereby enabling them to automatically process network information and seek network-related behavior in the unconscious state. Therefore, IAs may easily find and pay attention to network information in unconscious conditions. Second, according to Franken’s cognitive processing model^9^, the IAs’ increasing automatic detection of network information can improve the potential possibility of network behaviors, thereby indicating automatic detection advantage toward network information. Finally, according to the integration view of Field and Cox^53^, Internet images lead to positive expectations of network behaviors of IAs due to classical conditioning, in which the attention of IAs is automatically “grabbed” once the Internet-related picture appears.

Experiment 2 used the task-irrelevant deviant-standard reverse oddball paradigm. MMN was used as an index to investigate the difference in neural reactivity between the IAs and the control group when they automatically detected the characteristics of Internet-related and neutral pictures. Three interesting results were obtained. (1) The MMNs of the Internet-related pictures of the IAs were enhanced compared with those of the control group. (2) The MMN of the neutral pictures of the control group were enhanced compared with those of the IAs. (3) The MMNs of the red pictures were enhanced compared with those of the black pictures.

This study referred to Müller’s^40^ paradigm of characterizing different classes of objects under unconscious conditions. The paradigm makes the same color changes to two types of pictures. In this case, if the MMN induced is different, this difference in MMN is not triggered by color changes but the different individual detection sensitivities of the two types of pictures. The results of this study showed that IAs’ MMN induced by Internet-related pictures was significantly larger than it induced by neutral pictures (see Fig. 3a–c), suggesting that the automatic detection sensitivity of IAs toward Internet-related pictures was indeed greater than it toward neutral pictures. Although there has been no literature on the IAs’ automatic detection advantage toward Internet information yet, other addiction-related studies can indirectly support the findings of this study. For example, Petit^54^ found that alcohol cues induce the increase in N1 component among alcoholics. Versace^55^ found that cigarettes cues induce the increase in P1 component among cigarette addicts. Asmaro^56^ found that cannabis-related cues induce the increase in early positive component among cannabis addicts. Nijs *et al*.^57^ found that high-calorie food induces the increase in P2 component among obese individuals. Based on the dual-processing theory, D’Hondt and Maurage^58^ reviewed the electrophysiological research on Internet addiction. It was found that IAs’ activation of automatic-affective processing system was increased by Internet-related cues, while activation of reflective system was weakened. These researchers have believed that addicts have an early attentional processing advantage to addiction-related cues. The current study is the first to investigate the visual MMN enhancement effect on Internet-related cues of IAs and provides the most direct and reliable evidence of the automatic detection advantage toward network-related cues.

A model of conscious and unconscious processes in audition^21^ states that, the input of the auditory channel is first detected by feature; then, it enters sensory memory registration after feature integration, thereby forming a sensory memory template. If the feature has undergone an obvious change, then detection and integration will result in a refresh of the sensory memory, which is the mechanism of MMN production in the primary cortex of the auditory channel. Although Näätänen *et al*. did not propose a similar theory in the visual channel, they mentioned that visual MMN may present similar neural mechanisms. On the basis of this theory, we agreed that the first possible cause of increased MMN to Internet-related pictures among IAs is their tendency to detect the presence of changes in their sensory memory template formed by the Internet-related picture. The second possible reason is existence of specific cells for detecting the network characteristics of the Internet-related pictures that exist in the primary cortex of the IAs. Therefore, when the picture changes, the specific cells are sensitive and the mismatch of sensory memory after feature integration is obvious, thereby inducing increased MMN. The third reason is the incentive sensitization model^14,15,59^. IAs are reinforced by the pleasure of addictive behaviors, which change the brain function related to reward circuits. The motivation center gradually becomes sensitive to network information, thereby causing the IAs to express the characteristics of network information psychologically and implicitly through salience of incentives. Thus, the automatic detection of network information characteristics is continuously enhanced. The study of Balconi *et al*.^60^ supported this hypothesis. In their study, IAs’ EEGs were recorded when performing Go/NoGo tasks. Behavioral results showed that IAs responded significantly faster to Internet-related cues than to neutral cues. ERP results showed that the amplitude of FRN (Feedback Related Negativity) induced by Internet-related cues was significantly smaller than by neutral clue among IAs. However he P300 amplitude induced by Internet-related cues was significantly greater than by neutral cues. Therefore, they believed that IAs have higher motivational salience and reward expectations for Internet-related cues. The results of several fMRI studies supported this hypothesis indirectly. An abnormal function exists in the reward circuit related brain regions in the IAs and is manifested by the unusual activation in the relevant brain regions induced by network-related information^61,62^. This special processing advantage of IAs on Internet pictures may also be related to the structural atrophy of the motivation circuits (the orbitofrontal cortex)^63–65^. The orbitofrontal cortex can produce target orientation behaviors by assessing the importance of current stimuli and predicting possible outcomes. That is, IAs may have great amplitude of MMN on the Internet-related picture, possibly because their orbitofrontal cortexes are impaired, thereby leading to increased desire for network. Therefore, when the Internet-related picture appeared, the IAs experienced a strong motivation for cognitive processing and a large amount of attentional resources would be allocated to the Internet-related picture. Consequently, detecting changes in the Internet-related pictures was easy, thereby creating a significantly large MMN.

The findings of the study have two implications. First, although several studies have found that the IAs show attentional bias toward Internet-related cues^6,10,66^ and that Internet-related cues can induce the craving of IAs for Internet behaviors^7^, the investigation of the automatic detection advantage is insufficient^21,22^. This study has made up for this defect from two aspects. On the one hand, we prove IAs’ automatic detection advantage of Internet-related pictures from the neural level. On the other hand, the results of this study provide a neural explanation of the former findings that IAs have attentional bias to the Internet-related cues, which suggests that IAs’ cognitive nervous system may have neural dominance in network-related cues. That is, under the activation of addictive motivation, the neural system of IAs will automatically detect the network information in the environment.

As for the enhanced MMN of the control group compared with that of IAs on neutral pictures (see Fig. 3a, MMN of neutral pictures of the control group is greater than that of IAs) may be due to the impairment of the sensory processing capacity of IAs to the neutral material. Considerable research has found that schizophrenia^31,32^, depression^33–35^, long-term amphetamine abusers^36^, alcohol abusers^23,67^, and teenagers with reading disorder^68^ suffer impaired visual perception. Thus, the results may indicate that the IAs’ excessive attention to addiction-related cues leads to a decline in the perceived ability of visual non-internet information; therefore, their visual processing of other neutral events in their lives has been impaired. For example, Jiao *et al*.^69^ found that IAs’ empathy ability to pain images was impaired, showing a decrease in the amplitude of N1, N2 and P3. Another possible reason is that the sensitization of network-related information weakens the IAs’ arousing to the non-network information (natural reward or neutral cues). A study of substance addiction indicated that the declined arousal response and the lack of pleasure to the natural reward cues may lead to addicts’ excessive reaction to addictive cues, and the Internet addiction behaviors will be used to compensate for this lack to obtain pleasure^70^. Therefore, the IAs may also show a decrease in the response to natural reward cues and a lack of pleasant sensation. The neutral cues used in this study were pictures of common objects in life. Since the IAs may be less likely to wake up to natural reward cues, no natural concern and arousal for this daily living equipment will be induced. Few PET and fMRI studies on Internet addiction have provided circumstantial evidence for this view, as they found that the baseline metabolism and dopamine receptor levels in reward circuits are significantly lower when the IAs are without Internet information input^71–73^. This deduction explains the declined arousal level and depression of IAs in the absence of Internet and why the IAs have very small MMN amplitudes induced by neutral pictures.

Another interesting finding of this study was that red pictures could induce greater MMN than black pictures (see Fig. 3a,c, MMN of red pictures are greater than that of black pictures among IAs and the control group), suggesting that both the IAs and the control group are more sensitive to red pictures and more easily detect their changes. Previous studies have shown that long wave colors (such as red) are emotionally arousing, suggesting that red can induce high arousal emotions^74,75^. According to learned association theory, we have been experiencing implicit or explicit associations between color and various information since infancy, which results in color connection after repetition. Hence, when we see the corresponding color in a certain environment, we spontaneously activate the learned association of the color, which affects our mental activities^76^. Thus, we are more sensitive to red than black pictures. Gao^77^ used Neuropock-8 visually evoked potential apparatus and found that, at the same brightness, our vision reacts more sensitively to colored squares than to black and white squares, that is, the incubation period is slightly small. Studies have also shown that, in monochrome, the sensitivity of the eye to red is second only to that of blue and green^78^. These studies have provided direct evidence for subjects who are sensitive to red pictures. In addition, red presents a positive significance for Chinese students^79^. As in festivals, red symbolizes happiness and auspiciousness. Meanwhile, politically, red represents revolution, justice, courage, and hope, among others. Thus, red can lead to the arousal of individuals and thus make them sensitive to the change in the red picture.

There were several limitations of the current study. First, the selection of the color of the stimulus is not optimal. In this study, we used color change to control the unconscious and automatic processing degree of the subjects, but the choice of color type was inappropriate to some extent because red and black were not in the same color system. Red is in a chromatic color system while black is in a neutral color system, for which red color is obviously more noticeable than black which is incomparable. Therefore, it is better to choose two colors in the same system, such as red and green, if future experiments continue to use color type as a control variable. Second, the sample size in this study was kind of small, which might reduce the power of the statistical analyses and hamper generalization of the findings.

In summary, the automatic detection of network information of the IAs presents advantages in behavioral and neural responses. The behavioral and MMN indexes of the superiority effect can be used as an objective basis for detecting Internet addiction.

## Methods and Materials

### Behavioral Experiment

#### Subjects

A random sampling survey of undergraduates in three universities in Wuhan was conducted using the Internet Addiction Test (IAT)^1^. A total of 900 questionnaires were collected and 840 valid questionnaires were returned. On the basis of the IAT diagnostic criteria (≥80points), 26 people (24 males and 2 females) with severe Internet addiction were selected. A total of 15 male addicts were randomly selected as the addicts group and 15 control individuals (of matching sex, age, profession, and so on) were selected from the survey sample as the control group. All subjects were right-handed, had normal or corrected visual acuity, had no parachromatoblepsia or hypochromatopsia, and had no history of mental illness or medication history of the central nervous system. They signed an informed consent form. All participants were compensated for taking part in this study. This study was approved by the institutional ethical committee of National Key Laboratory of Cognitive Neuroscience and Learning of China. We confirm that all methods were performed in accordance with the relevant guidelines and regulations. The subject information is shown in Table 2.

**Table 2:** Subject Information.

	Number	Age (M ± SD)	Internet use history (years)(M ± SD)	Daily Internet use (hour)(M ± SD)	IAT score (M ± SD)
IAs group	15	20.80 ± 1.01	9.27 ± 1.28	3.93 ± 0.65	83.20 ± 2.37
Control group	15	20.87 ± 1.06	9.47 ± 1.30	3.33 ± 0.49	27.33 ± 3.27
t		0.17	0.42	2.40	53.65
p		0.86	0.68	0.03*	0.00**

### Materials and Procedure

Experimental materials were produced through a questionnaire. Internet-related pictures were made from a few commonly used network logos, and the neutral pictures were the most common things in life for balancing familiarity. We first removed the background information and made the backgrounds white and the outline black for these two types of pictures. Then, we extracted the outlines of the network logos and objects. Eliminating the irrelevant elements such as background and color, only the main contour lines that represent the characteristics of the network were retained. The schematic diagrams then were turned into black-and -white pictures. The size and resolution of the pictures were adjusted to the same. Finally, 25 Internet-related and 25 neutral pictures were obtained. Subsequently, 20 college students were asked to evaluate the two attributes of the experimental materials. The first property is the picture category, which is either a Internet-related or a neutral class. The second attribute is familiarity, which is scored using a five-point Likert scale (very familiar – 5 points, familiar – 4 points, moderately familiar – 3 points, not very familiar – 2 points, not familiar at all, 1 point). Finally, 10 Internet-related and neutral pictures, which are completely consistent with the result of a classified evaluation, were taken as the official experimental materials. Paired samples t test of the average familiarity showed that there was no significant difference (*t*_(19)_ = 1.52, p = 0.15) between Internet-related (4.44 ± 0.51) and neutral pictures (4.20 ± 0.52).

The stimuli were rendered using the visual dot-probe paradigm with masking. The experiment was performed on a desktop computer with a screen size of 15 inches. The PC host model was DELL OPTIPLEX 745, and the graphoscope was Philips 202F4 CRT. The horizontal-scanning frequency was 30–115 KHz, the vertical scanning frequency was 50–160 Hz, and the bandwidth was 300 MHz. E-prime 2 software was used to prepare and present the experimental program. A fixation point “+” was shown at the center of the screen for 1000 ms first. Then, a pair of pictures on both sides of “+” was presented (Internet-related and neutral pictures appeared randomly). The picture size was 150 × 150 (pixels) and the resolution was 256 × 256 (dpi). The screen background was white. The visual angle was 3.68° × 3.42° and the visual distance was 70 cm. Internet-related and neutral pictures appeared on both sides of “+” and disappeared after 14 ms. Immediately, a pair of masking stimuli followed (meaningless pictures of pure colors of equal size as pictures) for 14 ms. After the masking stimuli had disappeared, a probe dot on the Internet-related or neutral picture position on both sides of the original “+” was presented. The subjects were asked to press the direction of the probe dot (left or right) as soon as possible. They were asked to press “F” for left and “J” for right. After pressing, the probe point disappeared. After the 500 ms interval, the center of the screen appeared again “+” and a new trial was duplicated. The button response time was limited to 2000 ms. If the button was not pressed in 2000 ms, then it would automatically enter the next trial. The trials during which the probe point appeared in the same position as the Internet-related picture were called congruent trials. The trials during which the probe point appeared in a different position from the Internet-related picture (in the same position as the neutral picture) were called incongruent trials. The response times to the congruent and incongruent trials were compared to investigate the automatic detection advantage of IAs toward the presence of Internet-related pictures^42^.

### ERP Study

The subjects were the same as those in Experiment 1. The interval between the two experiments was more than one month to reduce experimental pollution.

### Materials and Procedure

The black outline lines of Experiment 1 materials were turned into red, thereby producing two series of materials of black and red pictures.

**Figure 4:**
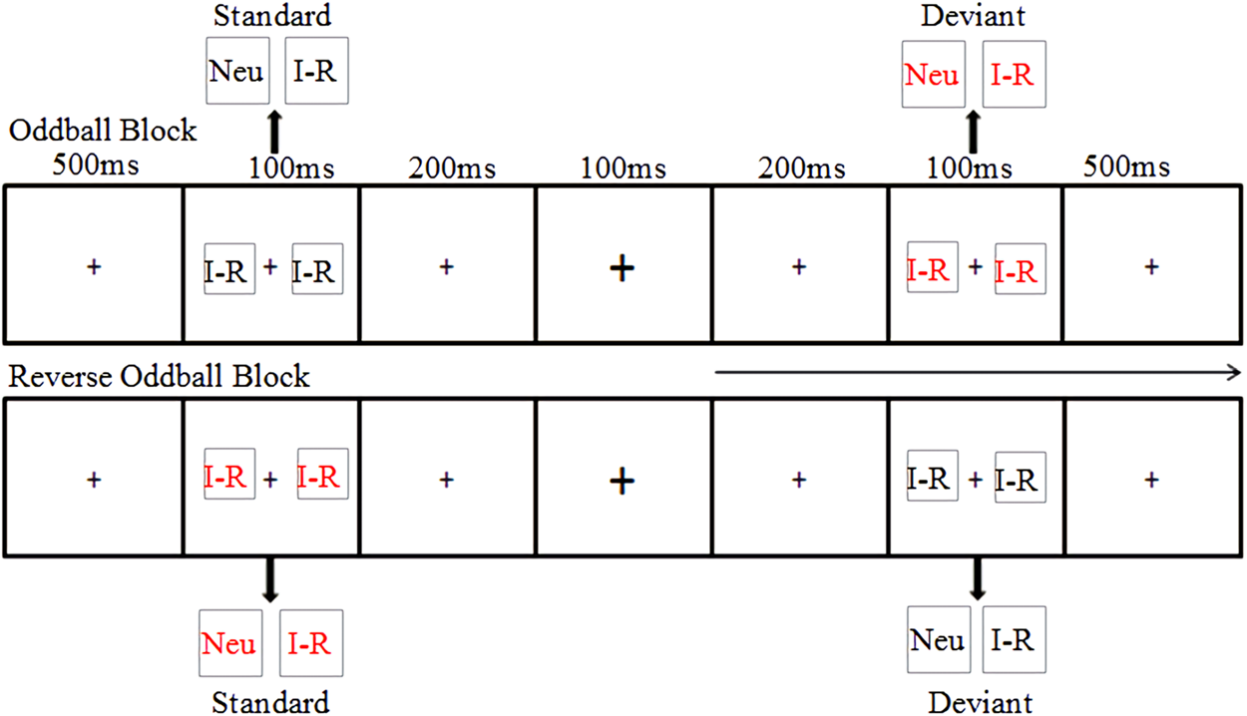
The scheme of the trial procedure. I-R, Internet-related pictures; Neu, Neutral pictures.

The scheme of the trial procedure was showed in Fig. 4. The Internet-related and neutral pictures appeared on both sides of the “+”. The picture rendering parameters were the same as those in Experiment 1, but the pictures were presented for 100 ms. “+” at the center of the screen turned larger or smaller at a random manner (mean frequency: 15/min, 66/block). The subjects were asked to ignore the stimulus on both sides of “+” and pay attention to judging the change in “+” at the screen and press the button as soon as possible. They were also asked to press “F” when “+” becomes larger and “J” when it becomes smaller.

The deviant-standard reverse oddball paradigm was used in the experiment. Four types of pictures (red Internet-related, black Internet-related, red neutral, and black neutral picture) were used as standard and deviant stimuli. To eliminate the possible interference caused by the physical attributes of the picture to the MMN, the same picture’s standard ERP was subtracted from the deviant ERP^28,80^. In this approach, each subject should complete four blocks. In block 1, black Internet-related pictures were used as standard stimuli and red Internet-related pictures as deviant stimuli. In block 2, red Internet-related pictures were used as standard stimuli and black Internet-related pictures were used as deviant stimuli. In block 3, black neutral pictures were used as standard stimuli and red neutral pictures were used as deviant stimuli. In block 4, red neutral pictures were used as standard stimuli and black neutral pictures were used as deviant stimuli. Each block contained 480 trials, during which 10 standard stimuli were first presented to establish a sensory memory pattern and then standard and deviant stimuli were presented in a pseudorandom series (probability was 8:2). Not less than two standard stimuli were presented between two deviations. The order of the four blocks was random between the subjects.

### ERP Recording and Analysis

ERP was continuously recorded (band pass 0.05–100 Hz, sampling rate 500 Hz) using a NeuroScan-64-guided EEG recording and analyzing system and referenced to the tip of the nose. The ground point was the midpoint of the FCz and Fz connections. VEOG and HEOG were recorded with two pairs of electrodes, one of which was placed above and below the right eye, and the other 10 mm from the lateral canthi. Electrode impedance was maintained below 5 kΩ throughout the experiment. The results were analyzed off-line. In the analysis, we removed four trials before and after the “+” changes to eliminate the disturbance of EEG components caused by the change in “+”. EOG artifacts were corrected using the method proposed by Semlitsch *et al*.^81^. The EEG was digitally filtered with a low-pass filter at 30 Hz (24 dB/Octave) and segmented in epochs of 600 ms (including a −100 ms to 500 ms pre-stimulus baseline). The ERPs greater than ± 75 μV were removed as artifact. The 8 experimental conditions (picture type 2 × color type 2 × deviant group – standard group 2) were superimposed on the average. In this manner, each subject produced 8 ERP averages. Four MMNs were produced by subtracting the deviant and standard ERPs of each subject. The results showed that the ERP received by all subjects under each condition was higher than 60 times that in trials, and no significant difference was observed in the number of ERPs accepted by the trials between the stimulus conditions.

In accordance with previous visual MMN literature and the ERPs’ total average in this study, O1 and O2 were selected as the representative electrodes of the left and right brain of the occipital region and PO5 and PO6 were the representative electrodes of the left and right brain in the temporal occipital region. The MMNs of all conditions were concentrated in the range of 200–300 ms. Therefore, the average amplitude of MMN in this period was measured and subjected to the four-way repeated-measure ANOVAs with subject group (IAs, control group), picture type (Internet-related pictures, neutral pictures), color type (red, black), and hemisphere (left, right). When appropriate, the degrees of freedom were corrected using the Greenhouse–Geisser estimate^82^.

### Data Availability

The datasets generated during and/or analysed during the current study are available from the corresponding author on reasonable request.

## References

[1] Young KS (1998). Internet addiction: The emergence of a new clinical disorder. Cyberpsychol behav..

[2] Young KS (1999). Internet addiction: symptoms, evaluation and treatment. Innovations in Clinical practice: A source book.

[3] He JB, Liu CJ, Guo YY, Zhao L (2011). Deficits in early-stage face perception in excessive internet users. Cyberpsychol Behav Soc Netw..

[4] APA. Diagnostic and Statistical Manual of Mental Disorders, 5th Edn. Washington, DC (2013).

[5] Griffiths, M. D., Kuss, D. J. & Demetrovics, Z. Social NetworkingAddiction: An Overview of Preliminary Findings[M]// Behavioral Addictions. *Elsevier Inc*. 119–141 (2014).

[6] Metcalf O, Pammer K (2011). Attentional bias in excessive massively multiplayer online role-playing gamers using a modified stroop task. Comput Hum Behav..

[7] Niu GF (2016). Cue-induced craving for internet among internet addicts. Addict Behav..

[8] Zhang, Y. *et al*. Brain activity toward gaming-related cues in internet gaming disorder during an addiction stroop task. *Front Psychol*. **7**, 10.3389/fpsyg.2016.00714 (2016). 10.3389/fpsyg.2016.00714 PMC4872468 27242623

[9] Franken IHA (2003). Drug craving and addiction: Integrating psychological and neuropsychopharmacological approaches. Prog Neuro-Psychoph..

[10] Holst RJ (2012). Attentional bias and disinhibition toward gaming cues are related to problem gaming in male adolescents. J Adolescent Health..

[11] Dai S, Ma Q, Wang X (2011). Attentional bias to addiction-related stimuli in internet addiction patients: an erp study. J Psychol Sci..

[12] Lu, J. *et al*. The brain functional state of music creation: an fMRI study of composers. *Sci Rep*. **5**, 10.1038/srep12277 (2015). 10.1038/srep12277 PMC4512184 26203921

[13] Tiffany ST (1990). A. cognitive model of drug urges and drug-use behavior: role of automatic and nonautomatic processes. Psychol Rev..

[14] Robinson TE, Berridge KC (1993). The neural basis of drug craving: an incentive-sensitization theory of addiction. Brain Res Rev..

[15] Robinson TE, Berridge KC (2000). The psychology and neurobiology of addiction: An incentive-sensitization view. Addiction..

[16] Mogg K, Bradley BP (1999). Orienting of attention to threatening facial expressions presented under conditions of restricted awareness. Cognition Emotion..

[17] Mogg K, Bradley BP (2002). Selective orienting of attention to masked threat faces in social anxiety. Behav Res Ther..

[18] Bradley B, Field M, Mogg K, De HJ (2004). Attentional and evaluative biases for smoking cues in nicotine dependence: component processes of biases in visual orienting. Behav Pharmacol..

[19] Constantinou N (2010). Attentional bias, inhibitory control and acute stress in current and former opiate addicts. Drug Alcohol Depen..

[20] Näätänen R, Gaillard AW, Mäntysalo S (1978). Early selective-attention effect on evoked potential reinterpreted. Acta Psychol..

[21] Näätänen R, Kujala T, Winkler I (2011). Auditory processing that leads to conscious perception: A unique window to central auditory processing opened by the mismatch negativity and related responses. Psychophysilogy..

[22] Czigler I, Balázs L, Winkler I (2002). Memory-based detection of task-irrelevant visual changes. Psychophysiology..

[23] He JB (2014). Different effects of alcohol on automatic detection of colour, location and time change: a mismatch negativity study. J Psychopharmacol..

[24] Kimura M, Katayama JI, Ohira H, Schröger E (2009). Visual mismatch negativity: new evidence from the equiprobable paradigm. Psychophysiology..

[25] Amenedo E, Pazo–Alvarez P, Cadaveira F (2007). Vertical asymmetries in pre-attentive detection of changes in motion direction. Int J Psychophysiol..

[26] Müller D (2010). Visual object representations can be formed outside the focus of voluntary attention: evidence from event-related brain potentials. J Cognitive Neurosci..

[27] Li, X. *et al*. Visual mismatch negativity elicited by facial expressions: new evidence from the equiprobable paradigm. *Behav Brain Funct*. **8**, 10.1186/1744-9081-8-7 (2012). 10.1186/1744-9081-8-7 PMC3292984 22300600

[28] Wang W, Miao D, Zhao L (2014). Automatic detection of orientation changes of faces versus non-face objects: a visual mmn study. Biol Psychol..

[29] Stefanics G, Czigler I (2012). Automatic prediction error responses to hands with unexpected laterality: an electrophysiological study. Neuroimage..

[30] Kecskés-Kovács SI, Czigler I (2013). Gender of faces is automatically detected: a visual mismatch negativity study. Front Hum Neurosci..

[31] Farkas K, Stefanics G, Marosi C, Csukly G (2015). Elementary sensory deficits in schizophrenia indexed by impaired visual mismatch negativity. Schizophr Res..

[32] Neuhaus AH (2013). Evidence for impaired visual prediction error in schizophrenia. Schizophr Res..

[33] Maekawa, T. *et al*. Altered visual information processing systems in bipolar disorder: evidence from visual MMN and P3. *Front Hum Neurosci*. **7**, 10.3389/fnhum.2013.00403 (2013). 10.3389/fnhum.2013.00403 PMC3724050 23898256

[34] Qiu X (2011). Impairment in processing visual information at the preattentive stage in patients with a major depressive disorder: a visual mismatch negativity study. Neurosci Lett..

[35] Chang Y, Xu J, Shi N, Zhang B, Zhao L (2010). Dysfunction of processing task-irrelevant emotional faces in major depressive disorder patients revealed by expression-related visual MMN. Neurosci Lett..

[36] Hosák L, Kremlacek J, Kuba M, Libiger J, Cizek J (2008). Mismatch negativity in methamphetamine dependence: a pilot study. Acta Neurobiol Exp..

[37] Kremláček J (2016). Visual mismatch negativity (vMMN): A review and meta-analysis of studies in psychiatric and neurological disorders. Cortex..

[38] Yang X (2016). Gender differences in pre-attentive change detection for visual but not auditory stimuli. Clin Neurophysilo..

[39] Wang, X. D., Wu, Y. Y., Liu, A. & Wang, P. Spatio-temporal dynamics of automatic processing of phonological information in visual words. *Sci Rep*. **3**, 10.1038/srep03485 (2013). 10.1038/srep03485 PMC6506442 24336606

[40] Mogg K, Bradley BP, Hallowell N (1994). Attentional bias to threat: Roles of trait anxiety, stressful events, and awareness. Q J Exp Psychol..

[41] Yan X (2009). Preconscious attentional bias in cigarette smokers: a probe into awareness modulation on attentional bias. Addict Biol..

[42] Noël X (2006). Time course of attention for alcohol cues in abstinent alcoholic patients: the role of initial orienting. Alcohol Clin Exp Res..

[43] Loeber S (2009). Clinical study: attentional bias in alcohol–dependent patients: the role of chronicity and executive functioning. Addict Biol..

[44] Vollstädtklein S, Loeber S, Von GC, Mann K, Kiefer F (2009). Avoidance of alcohol-related stimuli increases during the early stage of abstinence in alcohol-dependent patients. Alcohol Alcoholism..

[45] Ciccarelli M, Nigro G, Griffiths MD, Cosenza M, D’Olimpio F (2016). Attentional biases in problem and non-problem gamblers. J Affect Disorders..

[46] Rosse RB (1997). Preattentive and attentive eye movements during visual scanning of a cocaine cue: correlation with intensity of cocaine cravings. J Neuropsych Clin N..

[47] Ceballos NA, Komogortsev OV, Turner GM (2009). Ocular imaging of attentional bias among college students: automatic and controlled processing of alcohol-related scenes. J Stud Alcohol Drugs..

[48] Duijvenbode NV (2017). Attentional bias in problematic drinkers with and without mild to borderline intellectual disability. J Intellect Disabil Res..

[49] Castellanos EH (2009). Obese adults have visual attention bias for food cue images: evidence for altered reward system function. Int J Obesity..

[50] Werthmann J (2011). Can (not) take my eyes off it: Attention bias for food in overweight participants. Health Psychol..

[51] Brevers D (2011). Time course of attentional bias for gambling information in problem gambling. Psychol Addict Behav..

[52] Zhao H (2017). Eye Movement Evidence of Attentional Bias for Substance-Related Cues in Heroin Dependents on Methadone Maintenance Therapy. Subst Use Misuse..

[53] Field M, Cox WM (2008). Attentional bias in addictive behaviors: a review of its development, causes, and consequences. Drug Alcohol Depen..

[54] Petit G (2012). Early attentional modulation by alcohol-related cues in young binge drinkers: an event-related potentials study. Clin Neurophysiol..

[55] Versace F (2011). Brain reactivity to emotional, neutral and cigarette–related stimuli in smokers. Addict Biol..

[56] Asmaro D, Carolan PL, Liotti M (2014). Electrophysiological evidence of early attentional bias to drug-related pictures in chronic cannabis users. Addict Behav..

[57] Nijs IM, Franken IH, Muris P (2010). Food-related Stroop interference in obese and normal-weight individuals: Behavioral and electrophysiological indices. Eat Behav..

[58] D’Hondt F, Maurage P (2017). Electrophysiological studies in Internet addiction: A review within the dual-process framework. Addict Behav..

[59] Robinson TE, Berridge KC (2008). The incentive sensitization theory of addiction: some current issues. Philos T Roy Soc B..

[60] Balconi, M., Venturella, I., & Finocchiaro, R. Evidences from rewarding system, FRN and P300 effect in Internet-addiction in young people. *Brain Sci*. **7**, 10.3390/brainsci7070081 (2017). 10.3390/brainsci7070081 PMC5532594 28704978

[61] Ko CH (2011). Brain correlates of craving for online gaming under cue exposure in subjects with Internet gaming addiction and in remitted subjects. ADDICT BIOL..

[62] Sun Y (2012). Brain fMRI study of crave induced by cue pictures in online game addicts (male adolescents). Behav Brain Res..

[63] Yuan, K. *et al*. Cortical thickness abnormalities in late adolescence with online gaming addiction. *Plos ONE*. **8**, 10.1371/journal.pone.0053055 (2013). 10.1371/journal.pone.0053055 PMC3541375 23326379

[64] Weng C (2013). Gray matter and white matter abnormalities in online game addiction. Eur J Radiol..

[65] Jin C (2016). Abnormal prefrontal cortex resting state functional connectivity and severity of internet gaming disorder. Brain Imaging Behav..

[66] Jeromin F, Nyenhuis N, Barke A (2016). Attentional bias in excessive Internet gamers: Experimental investigations using an addiction Stroop and a visual probe. J Behav Addict..

[67] Kenemans JL, Hebly W, Van den Heuvel EHM, Grent-‘T-Jong T (2010). Moderate alcohol disrupts a mechanism for detection of rare events in human visual cortex. J Psychopharmacol..

[68] Wang JJ, Bi HY, Gao LQ, Wydell TN (2010). The visual magnocellular pathway in Chinese-speaking children with developmental dyslexia. Neuropsychologia..

[69] Jiao, C., Wang, T., Peng, X., & Cui, F. Impaired empathy processing in individuals with Internet addiction disorder: An event-related potential Study. *Front Hum Neurosci*, **11**, 10.3389/fnhum.2017 (2017). 10.3389/fnhum.2017.00498 PMC5649199 29085290

[70] Cheetham A, Allen NB, Yücel M, Lubman DI (2010). The role of affective dysregulation in drug addiction. Clin Psychol Rev..

[71] Kim SH (2011). Reduced striatal dopamine D2 receptors in people with Internet addiction. Neuroreport..

[72] Tian M (2014). PET imaging reveals brain functional changes in internet gaming disorder. Eur J Nucl Med Mol Imaging..

[73] Zhang Y (2015). Alterations in brain connectivity in three sub-regions of the anterior cingulate cortex in heroin-dependent individuals: Evidence from resting state fMRI. Neuroscience..

[74] Valdez P, Mehrabian A (1994). Effects of color on emotions. J Exp Psychol Gen..

[75] Wilson GD (1966). Arousal properties of red versus green. Percept Motor Skill..

[76] Baldwin MW, Meunier J (1999). The cued activation of attachment relational schemas. Soc Cognition..

[77] Gao S (1995). Reaction of color vision to spatial frequency and pattern recognition. J Southeast University..

[78] Zhao XM, Teng PC, Zong JG (2014). Study of Human Eye Visual Discrimination to Color-difference. Electron Sci & Technol..

[79] Huang XT, Huang W, Li XR (1991). The symbolic impication of colors to the Chinese. J Psychol Sci..

[80] Stefanics G, Csukly G, Komlósi S, Czobor P, Czigler I (2012). Processing of unattended facial emotions: A visual mismatch negativity study. Neuroimage..

[81] Semlitsch HV, Anderer P, Schuster P, Presslich O (1986). A solution for reliable and valid reduction of ocular artifacts, applied to the P300 ERP. Psychophysilogy..

[82] Czigler I (2014). Visual mismatch negativity and categorization. Brain Topogr..

